# Supplementary material for: Detection of dementia on voice recordings using deep learning: a Framingham Heart Study
Source: Alzheimers Res Ther. 2021 Aug 31;13:146. doi: 10.1186/s13195-021-00888-3 (PMC8409004; doi:10.1186/s13195-021-00888-3)
Supplement: Supplementary file 1 — Additional file 1: Table S1. Demographics of the participants who were non-demented (NDE, i.e., individuals with normal cognition (NC) and mild cognitive impairment (MCI)) at the time of the voice recordings. ApoE data was unavailable for one Gen 1 participant, thirteen Gen 2 participants, and one New Offspring Cohort (NOS) participant; MMSE data was not collected for all Gen 3, OmniGen 2, and NOS participants. [file 13195_2021_888_MOESM1_ESM.docx]

**Table S1**: Demographics of the participants who were non-demented (i.e., individuals with normal cognition and mild cognitive impairment) at the time of the voice recordings. ApoE data was unavailable for one Gen 1 participant, thirteen Gen 2 participants, and one New Offspring Spouse Cohort (NOS) participant; MMSE data was not collected for all Gen 3, OmniGen 2, and NOS participants.

| **Non-demented cases** | | | | | | |
| --- | --- | --- | --- | --- | --- | --- |
| **Cohort** | **N** | **Female** | **ApoE4+** | **Recordings** | **Age (years)** | **Mean MMSE** |
| Gen 1 | 89 | 60 | 15 | 160 | 91.2±3.1 | 26.7±2.3 |
| Gen 2 | 398 | 201 | 89 | 745 | 76.5±7.8 | 27.6±2.1 |
| Gen 3 | 5 | 1 | 0 | 8 | 60.4±10.6 | NA |
| OmniGen 1 | 12 | 3 | 3 | 16 | 72.2±8.1 | 26.0±2.5 |
| OmniGen 2 | 1 | 1 | 1 | 1 | 74.0±0.0 | NA |
| NOS | 2 | 1 | 0 | 4 | 86.5±5.3 | NA |
| Total | 507 | 267 | 108 | 934 | 78.8±9.3 | 27.4±2.2 |
