# Supplementary figures and images for: Detection of dementia on voice recordings using deep learning: a Framingham Heart Study
Source: Alzheimers Res Ther. 2021 Aug 31;13:146. doi: 10.1186/s13195-021-00888-3 (PMC8409004; doi:10.1186/s13195-021-00888-3)

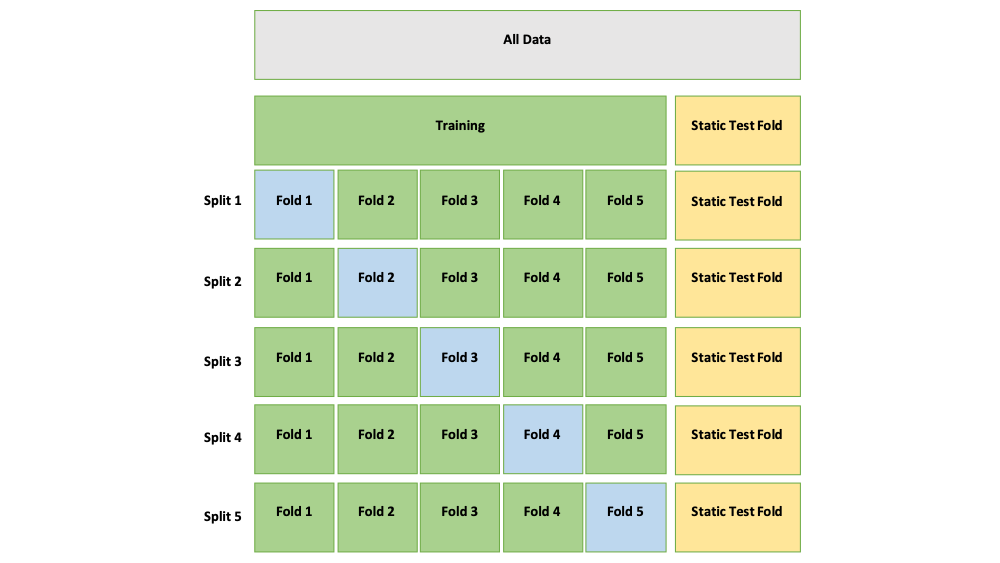

Supplement: Supplementary file 2 — Additional file 2: Figure S1. The dataset was first split into two parts such that a portion of the participants along with their recordings were kept aside for independent model testing. The models were trained on the remaining data using 5-fold cross-validation. We split the data on the participant level for each fold and then all of a given participant’s recordings were included in each fold. [file 13195_2021_888_MOESM2_ESM.tiff]

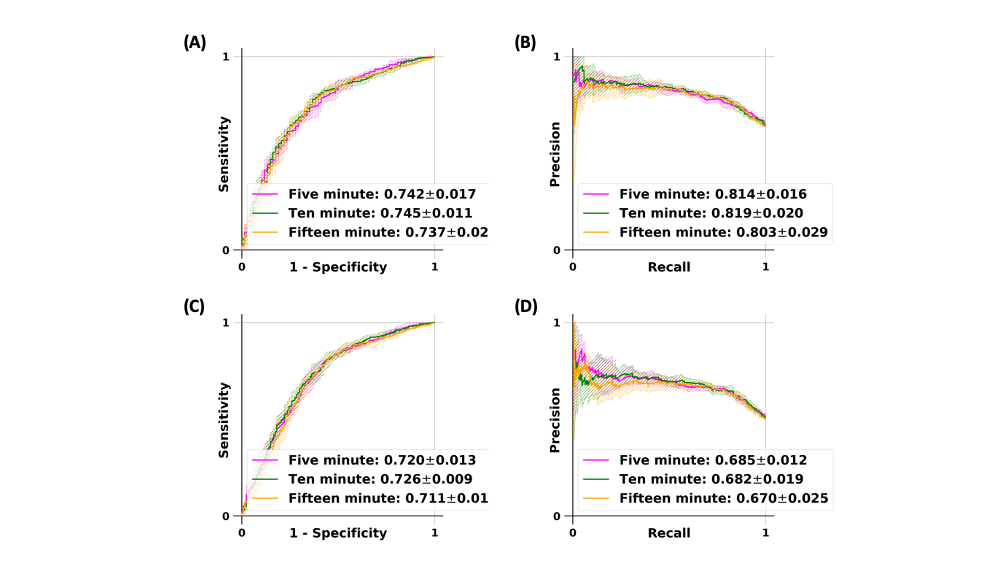

Supplement: Supplementary file 3 — Additional file 3: Figure S2. Long short-term memory (LSTM) networks were used to classify participants who have normal cognition from those with dementia and were used to classify participants who were not demented from those who were demented. The models were trained on full audio recordings and the performance was reported on audio samples of variable lengths extracted from the test data (see Figure S1). Plots (A) and (B) denote the ROC and PR curves for the LSTM model's performance on the normal cognition versus dementia task and plots (C) and (D) denote the ROC and PR curves for the LSTM model's performance on the non-demented versus demented task. [file 13195_2021_888_MOESM3_ESM.tiff]

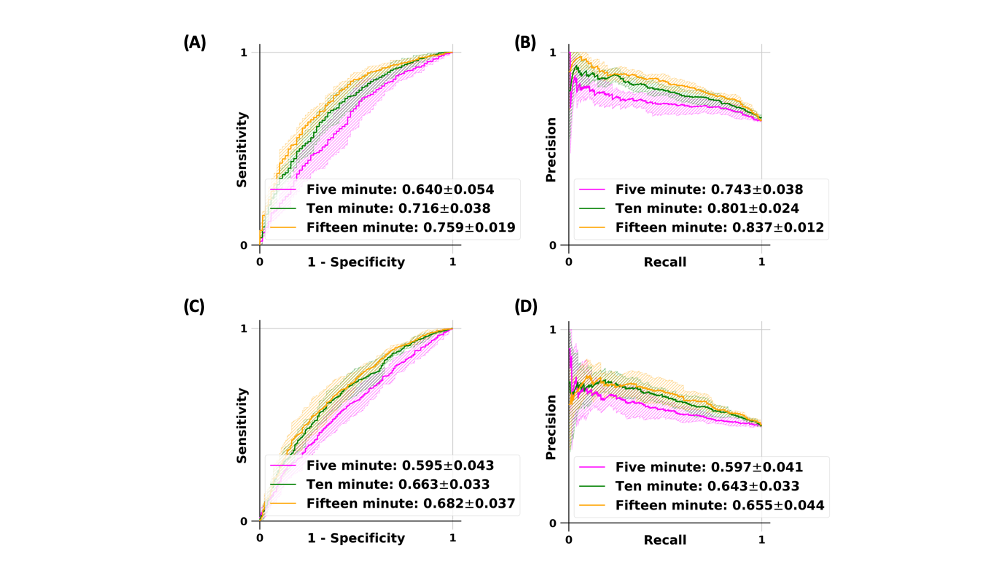

Supplement: Supplementary file 4 — Additional file 4: Figure S3. Convolutional neural network (CNN) models were used to classify participants who have normal cognition from those with dementia and were used to classify participants who were not demented from those who were demented. Models were trained on full audio recordings and the performance was reported on audio samples of variable lengths extracted from the test data (see Figure S1). Plots (A) and (B) denote the ROC and PR curves for the CNN model's performance on the normal cognition versus dementia task and plots (C) and (D) denote the ROC and PR curves for the CNN model's performance on the non-demented versus demented task. [file 13195_2021_888_MOESM4_ESM.tiff]
