# Supplementary material for: Detection of dementia on voice recordings using deep learning: a Framingham Heart Study
Source: Alzheimers Res Ther. 2021 Aug 31;13:146. doi: 10.1186/s13195-021-00888-3 (PMC8409004; doi:10.1186/s13195-021-00888-3)
Supplement: Supplementary file 5 — Additional file 5: Table S2. For the neuropsychological tests that have too few samples, the average salient administered fraction (SAF) and standard deviation for true positive (SAF[+]) and true negative (SAF[-]) cases are listed in descending order based on the SAF[+] value. SAF[+] is calculated by summing up the time spent in a given neuropsychological test that intersects with a segment of time that is DE[+] salient and dividing by the total time spent in a given neuropsychological test. SAF[-] is calculated by summing up the time spent in a given neuropsychological test that intersects with a segment of time that is not DE[+] salient and dividing by the total time spent in a given neuropsychological test. The number of samples for SAF[+] and SAF[-] indicate the number of true positive and true negative recordings that contain each neuropsychological test. [file 13195_2021_888_MOESM5_ESM.docx]

**Table S2:** For the neuropsychological tests that have too few samples and for the labeled segments that were unrelated to neuropsychological testing (Miscellaneous and Unlabeled), the average SAF (salient administered fraction) and standard deviation for true positive (SAF[+]) and true negative (SAF[-]) cases are listed in descending order based on the SAF[+] value. SAF[+] is calculated by summing up the time spent in a given neuropsychological test that intersects with a segment of time that is DE[+] salient and dividing by the total time spent in a given neuropsychological test. SAF[-] is calculated by summing up the time spent in a given neuropsychological test that intersects with a segment of time that is not DE[+] salient and dividing by the total time spent in a given neuropsychological test. The number of samples for SAF[+] and SAF[-] indicate the number of true positive and true negative recordings that contain each neuropsychological test. Segments of time were marked as “Miscellaneous” when there was speech in between neuropsychological tests that was unrelated to testing. Segments of time at the start of recordings up until speech occurred were considered “Unlabeled”.

| **Test** | **SAF[+]** | **SAF[-]** | **SAF[+] samples** | **SAF[-] samples** |
| --- | --- | --- | --- | --- |
| **Balance Physical Function Test** | 1.00±0.00 | 0.63±0.30 | 2 | 2 |
| **Logical Memory Delayed Recall (alternate prompt)** | 1.00±0.00 |  | 1 | 0 |
| **Digit Symbol Learning** | 0.77±0.41 | 0.81±0.33 | 6 | 3 |
| **Math Fluency** | 0.74±0.34 |  | 4 | 0 |
| **Digit Symbol Recall** | 0.74±0.46 | 0.40±0.53 | 3 | 3 |
| **Logical Memory Multiple Choice (alternate prompt)** | 0.64±0.00 |  | 1 | 0 |
| **Unlabeled** | 0.61±0.50 | 0.25±0.44 | 31 | 20 |
| **Miscellaneous** | 0.57±0.39 | 0.47±0.45 | 36 | 15 |
| **Logical Memory Immediate Recall (alternate prompt)** | 0.46±0.18 |  | 2 | 0 |
| **Digit Symbol Coding** | 0.43±0.43 | 1.00±0.00 | 9 | 3 |
